# Editorial: Community series in towards precision medicine for immune-mediated disorders: advances in using big data and artificial intelligence to understand heterogeneity in inflammatory responses, volume II

**DOI:** 10.3389/fimmu.2025.1553004

**Published:** 2025-01-13

**Authors:** Xu-jie Zhou, Yasmina Laouar, Lam C. Tsoi

**Affiliations:** ^1^ Renal Division, Peking University First Hospital, Beijing, China; ^2^ Kidney Genetics Center, Peking University Institute of Nephrology, Beijing, China; ^3^ Peking University Institute of Nephrology, Key Laboratory of Renal Disease, National Health Commission, Beijing, China; ^4^ Key Laboratory of Chronic Kidney Disease Prevention and Treatment (Peking University), Ministry of Education, Beijing, China; ^5^ State Key Laboratory of Vascular Homeostasis and Remodeling, Peking University, Beijing, China; ^6^ Department of Microbiology and Immunology, Michigan Medicine, Ann Arbor, MI, United States; ^7^ Department of Dermatology, Michigan Medicine, Ann Arbor, MI, United States; ^8^ Department of Biostatistics, University of Michigan, Ann Arbor, MI, United States; ^9^ Gilbert S. Omenn Department of Computational Medicine and Bioinformatics, Michigan Medicine, Ann Arbor, MI, United States; ^10^ Mary H Weiser Food Allergy Center, Michigan Medicine, Ann Arbor, MI, United States

**Keywords:** artificial intelligence, big data, heterogeneity, immune-mediated disorders, multi-omics, precision medicine

The advent of big data and artificial intelligence (AI) has ushered in a new era in understanding the complexities of immune-mediated disorders. This second volume of our Community Series continues to explore the frontier of precision medicine for these conditions, showcasing significant advancements in utilizing AI to comprehend inflammatory response heterogeneity and develop personalized treatment approaches (**Figure 1**).

**Figure 1:**
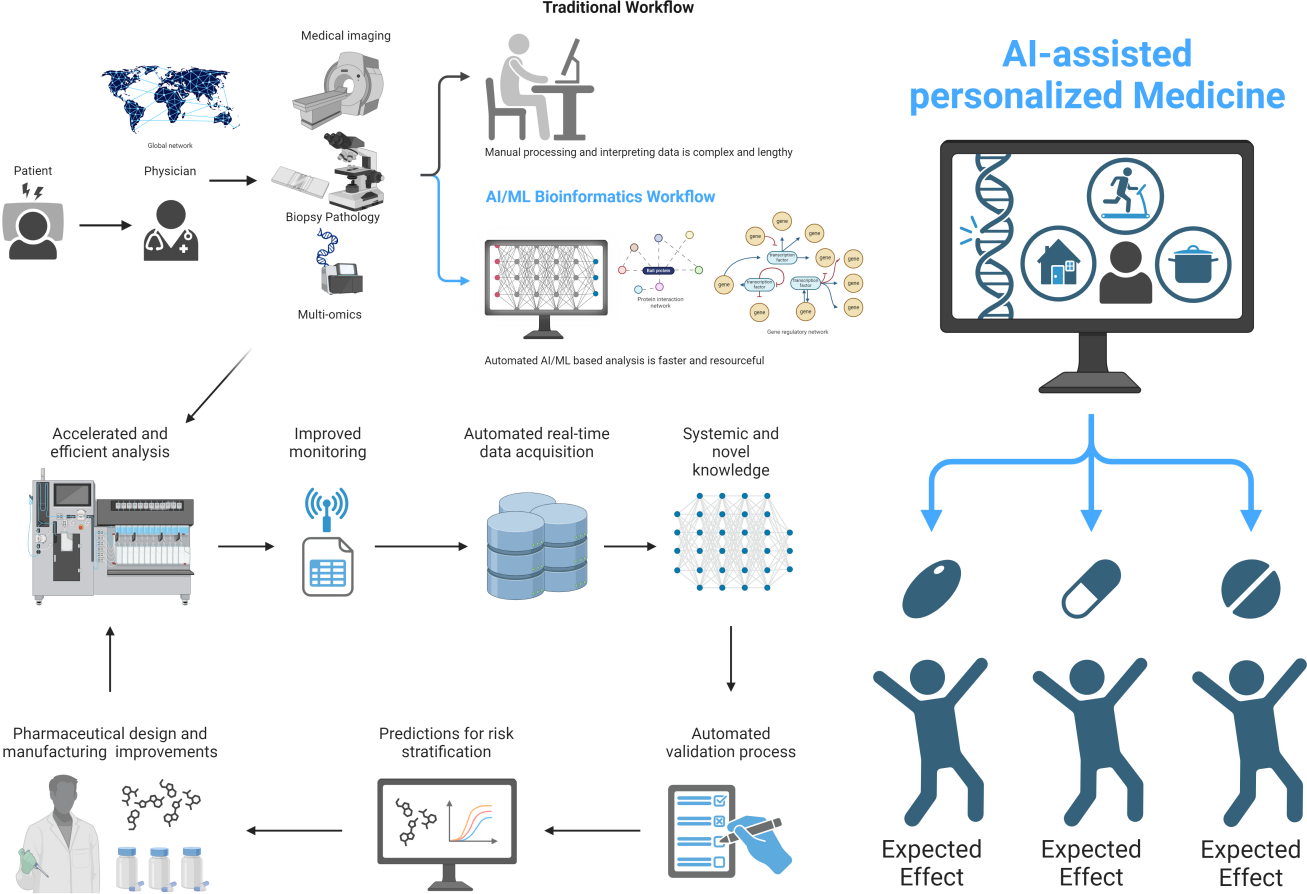
Significant advancements in utilizing AI to comprehend inflammatory response heterogeneity and develop personalized treatment approaches. This figure illustrates the transition from traditional workflow to AI-assisted personalized medicine in understanding and treating inflammatory responses. The traditional workflow is complex and time-consuming, involving manual processing and interpretation of data. AI-assisted methods can lead to more efficient and personalized approaches in medicine, particularly in the context of inflammatory responses. The expected effects of this transition include improved accuracy, faster processing times, and more tailored treatment strategies for individual patients.

AI has emerged as a powerful tool in deciphering immune system heterogeneity, offering unprecedented insights into the intricate networks of immune cells and their interactions (1, 2). Machine learning algorithms, particularly unsupervised learning techniques, have revolutionized our ability to analyze high-dimensional immunological data, identifying distinct cell populations and characterizing their phenotypes based on marker expression. This approach enhances our understanding of immune cell subsets and their roles in diseases, paving the way for more targeted therapeutic interventions.

## Machine learning for disease classification and prediction

Several studies in this Research Topic highlight the power of using machine learning (ML) to enhance disease classification and predict outcomes. Xu et al.’s work on predicting T-scores in IgA nephropathy using machine learning showcases the potential of AI in reducing the need for invasive kidney biopsies. This approach could significantly improve patient care by allowing for earlier diagnosis and more timely treatment initiation. The model’s ability to predict pathological severity using routine clinical characteristics offers a valuable tool for clinicians, especially in cases where kidney biopsy is not feasible or advisable. Future research may focus on prospective validation studies and the integration of multi-omics data to enhance the model’s predictive power.

Shi et al.’s application of machine learning in rheumatoid arthritis (RA) management represents a significant step towards personalized medicine. Their models for predicting treatment responses and disease progression could guide clinicians in selecting the most effective therapies for individual patients. This approach has the potential to optimize treatment outcomes, reduce adverse events, and improve overall patient care in RA management. Future development of user-friendly interfaces and decision support tools that seamlessly incorporate AI-derived insights into clinical practice may be of special importance.

Li et al.’s novel semi-supervised convolutional neural network (sscNOVA) for predicting functional regulatory variants in autoimmune diseases addresses the challenge of limited labeled data in genomics research. This innovative approach could accelerate the identification of disease-associated variants, potentially leading to improved risk prediction and the development of targeted therapies for autoimmune conditions.

## Radiomics and imaging biomarkers

The application of radiomics in ankylosing spondylitis (AS) by Hu et al. represents a significant advance in non-invasive disease phenotyping. By extracting quantitative features from MRI scans, this approach offers a more objective method for assessing hip involvement in AS. The potential for early detection of hip involvement could lead to more timely interventions, potentially slowing disease progression and improving patient outcomes. This study demonstrates the power of AI in extracting clinically relevant information from medical imaging data, potentially reducing the need for invasive diagnostic procedures.

## Large-scale genetic and epidemiological analytics

The comprehensive analysis of skin disease comorbidities by Li et al. provides valuable insights into shared pathophysiology and risk factors. This large-scale epidemiological study leverages big data to uncover patterns of disease co-occurrence, potentially guiding the development of comprehensive patient care strategies that take into account the interconnectedness of different immune-mediated conditions.

Several studies in this Research Topic employ Mendelian randomization (MR) to investigate causal relationships between immune-mediated disorders and various comorbidities (Su et al., Xie et al., Hu et al., Li and Liu, Ye et al., Bai et al.). The discovery of common genetic factors between type 1 diabetes and other autoimmune disorders may pave the way for the creation of treatments that can target multiple conditions concurrently. The investigation of drug targets for Sjögren’s syndrome through multi-omics MR and colocalization analyses offers a highly promising strategy for discovering new therapeutic targets^13^. The study on efgartigimod for refractory immune-mediated necrotizing myopathy highlights the potential of targeted therapies in managing challenging immune-mediated disorders (Yang et al.). This work demonstrates the importance of translating insights from basic immunology research into clinical practice, offering hope for patients with difficult-to-treat conditions. These studies provide robust evidence for shared genetic factors and potential causal pathways, which could inform drug repurposing efforts and the development of novel therapeutic strategies. The use of MR in these studies demonstrates the power of combining genetic data with advanced statistical techniques to uncover causal relationships that may not be apparent through traditional observational studies.

## Clinical implications and translational value

The research presented in this Research Topic demonstrates the immense potential of big data and AI in advancing precision medicine for immune-mediated disorders. From improving diagnostic accuracy and treatment selection to uncovering novel drug targets and causal relationships, these studies offer a glimpse into the future of personalized healthcare.

The ML and radiomics approaches presented have the potential to significantly enhance diagnostic accuracy and enable earlier interventions. For example, the ability to predict T-scores in IgA nephropathy without invasive biopsies could lead to more timely treatment initiation and improved patient outcomes. Similarly, the radiomics-based phenotyping of hip involvement in AS could allow for earlier detection and management of this complication, potentially preventing long-term disability.

The ML models developed for RA management offer the promise of more personalized treatment strategies. By predicting individual patient responses to diverse therapies, these models have the potential to aid clinicians in selecting the most effective treatments, minimizing adverse events, and ultimately enhancing overall outcomes. This approach is consistent with the aims of precision medicine and could significantly elevate the quality of care for RA patients.

The large-scale epidemiological analysis of skin disease comorbidities provides a foundation for improved risk stratification. Clinicians could utilize this information to design tailored screening programs and preventive measures for patients with particular skin conditions, aiming to lower the occurrence of related comorbidities.

The MR studies in this Research Topic offer valuable insights into potential drug targets and repurposing opportunities. The discovery of common genetic factors between different autoimmune disorders may pave the way for the creation of treatments that can target multiple conditions concurrently, potentially revolutionizing the treatment landscape for immune-mediated diseases.

## Challenges and future directions

Despite these advances, significant challenges remain in translating AI-driven approaches into clinical practice. The development of standardized protocols for data collection, processing, and integration across multiple modalities (e.g., imaging, genomics, clinical data) is crucial for the widespread acceptance of AI-driven approaches. Moreover, enhancing the interpretability of complex AI models is essential for fostering trust among clinicians and enabling their seamless integration into clinical decision-making frameworks.

To address these challenges, future research should focus on: *i)* Developing robust, externally validated AI models that incorporate diverse data types and account for population heterogeneity. *ii)* Creating interpretable AI algorithms that provide clinicians with clear rationales for their predictions and recommendations. *iii)* Designing user-friendly interfaces and clinical decision support tools that integrate AI-derived insights into existing clinical workflows. *iv)* Conducting large-scale, prospective clinical trials to demonstrate the real-world impact of AI-driven approaches on patient outcomes. *v)* Exploring the potential of federated learning and other privacy-preserving techniques to enable collaborative research while protecting patient data.

As we look forward to the third volume of this series, we anticipate studies that address these challenges and push the boundaries of AI applications in immunology. We further encourage submissions that integrate multi-omics data with clinical information to develop more comprehensive predictive models, explore the use of explainable AI techniques to enhance the interpretability of complex immunological models, and investigate the application of AI in real-time monitoring and prediction of immune responses, particularly in the context of immunotherapies. Additionally, we welcome research that develops AI-driven approaches for personalized vaccine design and optimization, as well as studies that explore the use of AI in deciphering the complex interactions between the immune system and the microbiome. These areas of focus represent exciting frontiers in the application of AI to immunology and have the potential to significantly advance our understanding and treatment of immune-mediated disorders.

In conclusion, the studies presented in this Research Topic demonstrate the immense potential of AI in advancing our understanding of immune-mediated disorders and developing personalized treatment approaches. By harnessing the power of big data and AI, we are moving closer to realizing the promise of precision medicine in immunology, ultimately improving patient outcomes and quality of life. The advancement of precision medicine in immune-mediated disorders is evident, driven by big data and AI. Through sustained investment in these methodologies and overcoming forthcoming obstacles, we envision a future where customized, efficacious therapies for immune-mediated disorders are commonplace, not rare instances.
